# Splenectomy modulates early immuno-inflammatory responses to trauma-hemorrhage and protects mice against secondary sepsis

**DOI:** 10.1038/s41598-018-33232-1

**Published:** 2018-10-05

**Authors:** S. Drechsler, J. Zipperle, P. Rademann, M. Jafarmadar, A. Klotz, S. Bahrami, M. F. Osuchowski

**Affiliations:** 1 Ludwig Boltzmann Institute for Experimental and Clinical Traumatology in the AUVA Research Center, Vienna, Austria; 2 Present Address: Center for Experimental Medicine, Medical Faculty, University of Cologne, Cologne, Germany

## Abstract

In polytrauma patients, the impact of splenectomy is equivocal, ranging from negative to protective. We investigated the impact of splenectomy on immune responses in the 1^st^-hit polytrauma alone and on survival in the post-traumatic sepsis (2^nd^ hit). Female BALB/c mice underwent polytrauma (1^st^ hit) consisting of either a) TH: femur fracture, hemorrhagic shock or b) TSH: splenectomy, femur fracture, hemorrhagic shock. Additionally, the polytrauma hit was followed by cecal ligation and puncture (CLP) 48 h later and compared to CLP alone. Splenectomy improved the 28-day survival in secondary sepsis to 92% (from 62%), while TH lowered it to 46% (p < 0.05). The improved survival was concurrent with lower release of inflammatory cytokines (IL-6, CXCL-1, MCP-1) and increase of C5a post-CLP. In the polytrauma hit alone, TSH induced stronger neutrophilia (1.9 fold) and lymphocytosis (1.7 fold) when compared to TH mice. Moreover, TSH resulted in a 41% rise of regulatory T-cells and reduced the median fluorescence intensity of MHC-2 on monocytes by 55% within 48 h (p < 0.05). Conversely, leukocyte phagocytic capacity was significantly increased by 4-fold after TSH despite a similar M1/M2 macrophage profile in both groups. Summarizing, splenectomy provoked both immuno-suppressive and immuno-stimulatory responses but was life-saving in secondary sepsis. Additionally, the polytrauma components in 2-hit models should be tested for their effects on outcome; the presumed end-effect of the 1^st^ hit solely based on the common immuno-inflammatory parameters could be misleading.

## Introduction

Polytraumatized patients typically develop a systemic immune reaction, termed systemic inflammatory response syndrome (SIRS), accompanied by the compensatory anti-inflammatory response syndrome (CARS), which can result in a dysfunctional host immune response and predispose them for secondary infections and sepsis^1–3^. After the initial injury, the immune system is repeatedly challenged by emergency interventions such as aggressive fluid resuscitation, mechanical ventilation, acute damage control and/or delayed reconstructive surgeries^4^, which puts patients at risk for ultimate development of the persistent inflammation, immunosuppression and/or catabolism syndrome (PICS)^5^. The acute increase in susceptibility to septic complications is indeed mostly influenced by the mechanism and severity of injury and the number of injured organs/tissues^6,7^. Splenectomy is among the most frequently injured organs in trauma patients^8^ but clinical data on the effects of spleen injury/splenectomy in trauma are limited and equivocal. The surgical removal of the injured spleen seems to increase the risk for early^9^ and late infections with thromboembolic^10^ and immunologic consequences such as overwhelming post-splenectomy infection syndrome (OPSI)^11^. In fact, several relatively low-powered clinical studies observed that spleen preservation in trauma patients decreased the risk for pneumonia^12–15^.

Literature clearly shows that immunologic deregulations caused by traumatic injuries can be diverse. In regard to injury severity, in trauma patients with an Injury Severity Score (ISS) of at least 25, impairment of phagocytic capacity^16^ and decrease of Human Leukocyte Antigen D related (HLA-DR) receptor expression on monocytes^17^ were evident. In more severely injured patients (ISS > 35), an increase of regulatory T-cells (Tregs)^18^ and loss of T helper cells were reported^19^. However, strong immunosuppressive features coexisted with clear signs of post-traumatic immune activation; e.g. Sturm *et al*. showed a partially increased phagocytosis with simultaneously decreased antigen presenting capacity of PMNs in trauma patients (ISS > 28) compared to healthy controls^16^.

Significant protective impact of splenectomy has been shown in different murine disease models such as stroke^20^ and bacterial translocation after burn trauma^21^, in contrast to its detrimental effect on acute-kidney-mediated lung injury^22^. However, despite various attempts to establish reliable preclinical rodent models for polytrauma, none of them included the loss of the spleen; most frequently, the models combined hemorrhagic shock with either laparotomy^23,24^ or femur fracture^25–27^. A number of recent studies demonstrated that more severe polytrauma models including blunt chest trauma^28^, traumatic brain injury^29^ and burns^30^ appear to better recapitulate the immunologic and genomic responses observed in human polytrauma patients^31^. Their conclusions were typically based on results from *ex-vivo* tests^32–34^. Only few studies tested the relevance of the observed immunologic alterations in clinically relevant *in vivo* models that combined trauma with delayed secondary infection such as polymicrobial peritonitis and pneumonia^25,35–38^.

In this study, we investigated the effects of splenectomy as an added traumatic insult in a 2-hit mouse model of polytrauma (hemorrhagic shock with femur fracture) followed by abdominal sepsis after 48 hours. Our data show that additional splenectomy strongly improved long-term outcome after secondary septic insult by modifying various immuno-inflammatory responses after polytrauma.

## Materials and Methods

### Animals

3-month-old, inbred, female BALB/c mice (*Mus Musculus*) from Charles River Laboratories (Sulzfeld, Germany) were used for all experiments. All animals were delivered to our facility at least one week before the onset of the experiment. Mice were housed in groups of 5 animals per Type-III cage on a 12 h light-dark diurnal cycle with room temperature between 21 and 23 °C. Cages were enriched with carton houses, wooden boards, small blocks for gnawing as well as wood wool for nesting (Abbedd Lab & Vet Service, Vienna, Austria) to facilitate natural behavior prior to and throughout the experiments.

### Ethical statement

All animal procedures were approved by the Viennese (Austria) legislative committee (Animal Use Proposal Permission no: 343130/2013/14) and conducted according to the National Institutes of Health guidelines.

To ensure a comprehensive observation, all animals were checked by trained professionals (i.e. DVMs and/or trained personnel) at least 2 times per day once they entered the experiment. Given that the first hit (trauma) was sublethal, monitoring was more frequent (at least 3 times per day) after the induction of secondary sepsis to identify deteriorating animals and prevent them from suffering. To objectively classify the disease progress we established a score based on the mouse clinical assessment scoring system (M-CASS) suggested by Huet *et al*.^39^, including several clinical parameters of well-being such as posture, mobility, alertness, rapid weight gain, startle reflex and body temperature. Depending on increasing illness severity, 0, 1 or 2 points were selected for each parameter. Whenever an animal exceeded a score of eight, body temperature dropped to below 28 °C, (using a Fluke 52 Series II thermometer, Fluke, USA), or the righting reflex could no longer be triggered, the mouse was immediately euthanized. Mice were scored starting 24 h after CLP until day 5 or euthanasia. All surgical procedures were done under inhalation anesthesia (2–3% isoflurane, Forane®). For all experiments or at the end of the 28-day observation period mice were killed using deep inhalation anaesthesia with isoflurane followed by cervical dislocation.

### Experimental Design

**Figure 1:**
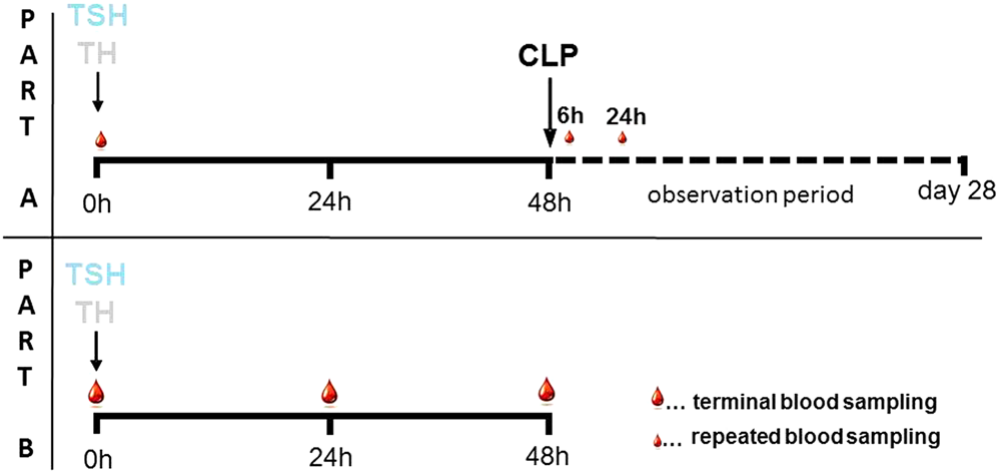
Experimental Study Design. (Part A):  12-week-old, female BALB/c mice were subjected to either TSH (femur fracture/splenectomy/hemorrhagic shock) or TH (femur fracture/hemorrhagic shock) followed by CLP (cecal ligation and puncture) to induce secondary sepsis 48 h later, or to CLP alone. 20 µl of blood, indicated by small blood drops, were collected at 6 h and 24 h post-CLP and survival was observed for 28 days. (Part B): 12 week old, female BALB/c mice were subjected to either TSH (unilateral femur fracture/splenectomy/hemorrhagic shock) or TH (unilateral femur fracture/hemorrhagic shock). Terminal blood, indicated by large blood drops, was collected either 24 h or 48 h post trauma.

The experiment was done in two parts. In part A, mice underwent either CLP alone (n = 22) or the 2-hit model consisting out of trauma (TSH or TH) followed by CLP 48 hours later (for TSH-CLP, n = 14 and for TH-CLP, n = 13). Survival was monitored for 28 days (Fig. 1A). In part B, a separate set of mice was subjected to either TH, consisting of unilateral femur fracture and hemorrhagic shock or TSH (unilateral femur fracture, splenectomy and hemorrhagic shock). 24 h and 48 h later mice were euthanized to collect heparinized whole blood and/or EDTA plasma for phagocytic activity, stimulation with LPS and further immune cell analyses.

### Trauma-hemorrhage model (TH)

The trauma-hemorrhage hit was identical to the first hit of our previously described 2-hit model^25^. Briefly, mice were fully anaesthetized using isoflurane (Forane®) inhalation and a unilateral non-comminuted fracture of the left femur with soft tissue-damage was caused by a custom-built forcipate device. Hemorrhagic shock was next induced by volume-based withdrawal of 40% of total blood volume (calculated as 6% of total blood volume) via retroorbital bleeding under additional local analgesia with oxybuprocainhydrochlorid (Novain®, Agepha, Austria). Mice were resuscitated subcutaneously with four times of the shed blood volume of Ringer’s solution, the first ¼ including analgesia (0.05 mg/kg buprenorphine, Bupaq®, Richter Pharma, Austria) was injected immediately post-TH, the remaining ¾ after 1 h.

### Trauma-hemorrhage with splenectomy model (TSH)

Femur fracture was induced as described above, subsequently followed by splenectomy performed as follows: left side of the abdomen was shaved and disinfected using betaisodona® solution, a 5 mm incision was made to open skin and abdominal cavity. The spleen was carefully exposed and the afferent and efferent vessels were ligated using Silkam® USP 4/0 before spleen was removed. Abdomen and skin were closed with single button sutures and Histoacryl® skin adhesive. The amount of blood removed via retroorbital puncture was reduced to 30% to compensate for the blood loss due to splenectomy. Resuscitation and analgesia were performed as described above (see trauma-hemorrhage model section).

### Cecal ligation and Puncture (CLP)

To induce polymicrobial peritonitis we subjected mice to cecal ligation and puncture surgery following the original protocol by Wichterman *et al*. with modifications specified elsewhere^25,40^. Shortly, mice were anaesthetized (2–3% isoflurane, Forane®), abdominal skin was shaved and disinfected, then the abdominal cavity was opened; cecum was exposed, ligated with Silkam 4.0 underneath the ileocecal valve and punctured twice with a 22 G needle to induce approximately 50% mortality (previously established in animals that underwent CLP alone). Abdomen was closed with single button sutures and skin was closed using Histoacryl® skin adhesive. All mice received analgesia (0.05 mg/kg buprenorphine, Bupaq®) prior to CLP. Starting 2 h after CLP, subcutaneous wide-range antibiotic therapy (25 mg/kg imipenem, Zienam®, MSD, Germany) and fluid resuscitation (0.5 ml Ringer’s solution) with analgesic (0.05 mg/kg buprenorphine, Bupaq®, Richter Pharma, Austria) was administered twice daily for five consecutive days post-CLP. Survival was followed for 28 days.

### Blood sampling

For terminal sampling, whole blood was removed via retroorbital puncture under deep inhalation anaesthesia (2–3% isoflurane, Forane®) and local analgesia (oxybuprocainhydrochlorid, Novain®, AGEPHA, Austria). In the survival study, starting at baseline (i.e. induction of TH/TSH), daily blood samples (20 µl) were drawn via facial vein (*vena submandibularis*) puncture with a 23 G needle at 6 h and 24 h post-CLP. All samples were collected in a pipette rinsed with ethylenediaminetetraacetic acid (K3-EDTA) and were immediately diluted 1:10 in PBS. After centrifugation (1000 × g, 5 min, 22 °C), 180 µl of diluted plasma was removed and stored at −80 °C for further analysis.

### Complete Blood Count

After removing plasma, the remaining blood pellet was resuspended with 180 µl Cell-Dyn buffer with EDTA and a complete blood count (CBC) with differential was performed with a CellDyn 3700 counter (Abbott Laboratories, Illinois, USA).

### Flow cytometry

A native pre-lysis staining procedure of EDTA-anticoagulated peripheral whole blood was employed to study the cellular immune status of the animals. Blood was diluted 1:2 with PBS and 100 µl of the suspension were transferred to FACS tubes (Greiner, Linz, Austria). Fluorophore-labelled anti-mouse antibodies against CD11b (FITC-conjugated), Ly6-G (PE-Cy5-conjugated), MHC-II (PE-conjugated), CD4 (FITC-conjugated), CD8a (PE-Cy7-conjugated), CD127 (PE-conjugated) and CD25 (PE-Cy5-conjugated) were purchased from eBioscience (Thermo Fisher, Waltham, MA, USA) and were diluted to appropriate working concentrations as provided by the manufacturer. Five microliters of the respective antibody dilution were added per tube and the sample was incubated at room temperature for 20 mins. Red blood cells (RBC) were disintegrated with a commercial multi-species RBC lysis buffer from eBioscience. After 2 washing steps with cold PBS, the pellet was resuspended in 300 µl of PBS and measured on a CytoflexTM flow cytometer (Beckman Coulter, Brea, CA, USA). Fluorophores were excited with a blue laser at 488 nm and emission was recorded through filtersets for the specific conjugate wavelengths. Samples were analyzed using the built-in Cytexpert 1.1 software from Beckman Coulter. Leukocyte subsets were identified by morphology in the forward-side scatter (FSC-SSC) and the positivity/negativity for the following antigens: granulocytes were defined as CD11b+Ly6Ghigh-SSChigh, and monocytes as CD11+Ly6Glow-SSClow. Expression of CD11b and MHC-II on the two subsets was monitored by recording the median fluorescence intensity of the respective antigens. Lymphocytes were defined by FSC-SSClow and regulatory T-cells were identified by CD4+CD25+ and the negativity for CD127. Representative examples of the gating strategies for monocytes and Tregs are demonstrated in Supplementary Fig. 1A,B.

### Flow cytometric assessment of macrophage polarization

Macrophage polarization was quantified by identifying circulating monocytic cells and by assessing the expression of either inducible NO-synthase (iNOS, NOS2) or the alternative activation towards Arginase I expression. Monocytic cells were gated based on the positivity for CD11b and MHCII and were further distinguished from granulocytes based on SSC and the negativity for Ly6G. Arginase I signal from the gated population was then blotted versus iNOS expression and polarization was defined as the directed expansion of the population towards prevalent iNOS or Arginase I expression. Events in the respective gate were calculated per measured volume and given as events/µL. The gating strategy is demonstrated in Supplementary Fig. 2.

### Cytokine Assay

Interleukin (IL)-1β, IL-10, IL-5, IL-6, interferon (IFN)-γ, tumor necrosis factor (TNF)–α, macrophage inflammatory protein (MIP)-1α, chemokine ligand (CXCL)-1 and monocyte chemoattractant protein-1 (MCP-1) were analyzed from plasma samples (6 h and 24 h post TSH-CLP/TH-CLP/CLP) using FlowCytomixTM Multiplex Kits (eBioscience, USA) according to the manufacturers protocol.

### Phagocytosis Assay

To assess phagocytic capacity of macrophages and granulocytes at 48 h after trauma, we used a modified pHrodo® Red *E. coli* BioParticles® flow cytometry assay (Thermo Fisher Scientific, Massachusetts, US). This kit utilizes *E. coli* fragments and a pH-sensitive conjugate with an excitation-emission characteristic similar to PE. Upon incorporation of the fragments in the phagolysosme of phagocytic cells, the transition to an acidic pH increases fluorescence intensity of the particles. Fifty µl of heparinized whole blood were transferred to FACS tubes and 10 µl of *E. coli* BioParticles® reagent were added to the sample. Together with the FITC-conjugated anti-mouse CD11b antibody, the samples were incubated on ice for 30 mins, lysed and washed as described in the flow cytometry section. Cells were identified by the positivity for the phagocytic Integrin CD11b and the presence of fluorescent bioparticles in the PE channel.

### *Ex vivo* LPS stimulation

For whole blood stimulation we used phenol extract from *E. coli* serotype O26:B6 (LPS, solution, Sigma Aldrich, USA) at a final concentration of 10 µg/ml. 350 µl blood (EDTA) were centrifuged for 12 min at 400 × g at room temperature. To adjust for the cell count, 110 µl from both plasma and cell pellet were collected and diluted with haematopoetic cell culture medium (RPMI-1640, Sigma Aldrich, St. Louis, MO, USA) in a 1:5 ratio. Next, either 20 µl of LPS solution or 0.9% saline was added to 480 µl of the previously prepared standardized blood. Samples were then incubated for 24 h at 37 °C and afterwards centrifuged (12 min at 1000 × g) prior to the quantification of synthetized cytokines with the FlowCytomixTM Multiplex Kit.

### C5a ELISA

Complement component C5a was assessed from mouse 1:10 diluted plasma samples using a standard enzyme linked immunosorbent assay (ELISA) kit (Cloud Clone Corp., Texas, US) according to the manufacturer’s instructions. The lower limit of detection was 1.56 ng/ml.

### Statistical analysis

The sample size estimations were calculated by power analysis with PS Power and Sample Size Calculations 3.0 prior to the study. We were planning a survival study with 1 control animal per experimental subject, an accrual interval of 0 days, and additional follow-up after the accrual interval of 28 days. Prior data indicated that the median survival time on the control treatment was 4 time units. If the true median survival times on the control and experimental treatments are 4 and 14 time units, respectively, we calculated that 11 experimental subjects and 11 control subjects are needed to be able to reject the null hypothesis that the experimental and control survival curves are equal with probability (power) 0.8. For the comparisons of immune responses after trauma we assumed a continuous response variable from independent control and experimental subjects with 1 control animal per experimental subject. Based on previous data on granulocyte counts we expected that each subject group would be normally distributed with standard deviation 600. If the true difference in the experimental and control means was 1000, 7 experimental subjects and 7 control subjects must be studied to be able to reject the null hypothesis that the population means of the experimental and control groups are equal with probability (power) 0.8. The Type I error probability associated of both tests test of this null hypothesis was 0.05.

Statistical analysis was performed using Graph Prism 5.01 (San Diego, California, US) Data were tested for normality using D’Agostino and Pearson and Shapiro-Wilk test. If necessary, data were transformed using a logarithmic transformation. For comparison between baseline and 24 h or 48 h time point we used 1-way ANOVA test with Bonferroni test for normally distributed data. For non-Gaussian distributed data after transformation, Kruskal Wallis Test with Dunn’s test for multiple comparisons was applied. Data are presented box and whiskers (min to max). 28-day survival curves were plotted using Kaplan-Meier method. Level of significance was set at p < 0.05.

## Results

The experiment was divided in Part A and B (Fig. 1). In part A, the role of splenectomy in the 2-hit model of posttraumatic sepsis was analyzed, while the effects of splenectomy on the immune response after trauma/hemorrhage were investigated in part B.

### Part A

#### Addition of splenectomy to the first-hit polytrauma attenuated mouse mortality in secondary sepsis

**Figure 2:**
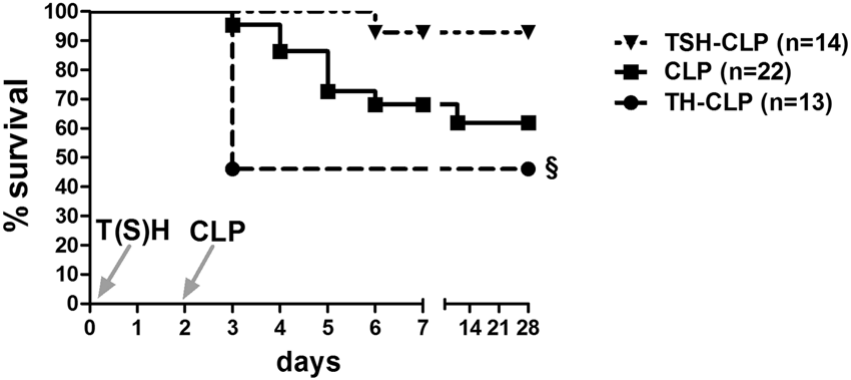
28-day Kaplan Meier Survival Curve. 3 month old, female BALB/c mice were subjected to femur fracture, splenectomy and hemorrhagic shock (TSH, triangle, n = 14) or femur fracture and hemorrhagic shock (TH, circle, n = 13) followed by cecal ligation and puncture (CLP) to induce polymicrobial abdominal sepsis 2 days later, or to CLP alone (square, n = 22). Survival was followed for 28 days. ^§^p < 0.05 compared to all other groups.

To uncover whether the cellular and functional changes of the innate and adaptive immunity caused by splenectomy in the polytrauma model affect the 28-day survival rate of secondary sepsis, mice were subjected either to a combination of TH or TSH with CLP 48 h later or to CLP alone (Fig. 1). Both trauma hits (TH and TSH) were sublethal within 48 hrs. Independent of the trauma model, deaths occurred mostly in the acute phase of sepsis (days 1–5). CLP alone resulted in 62% 28-day survival, but preceding TH worsened survival to 46% (p < 0.05 to all other groups). In contrast, additional splenectomy in the TSH hit resulted in a survival rate of 92% (p < 0.05 compared to TH-CLP) at day 28 post-CLP (Fig. 2).

#### Additional splenectomy dampened the inflammatory response in secondary sepsis

**Figure 3:**
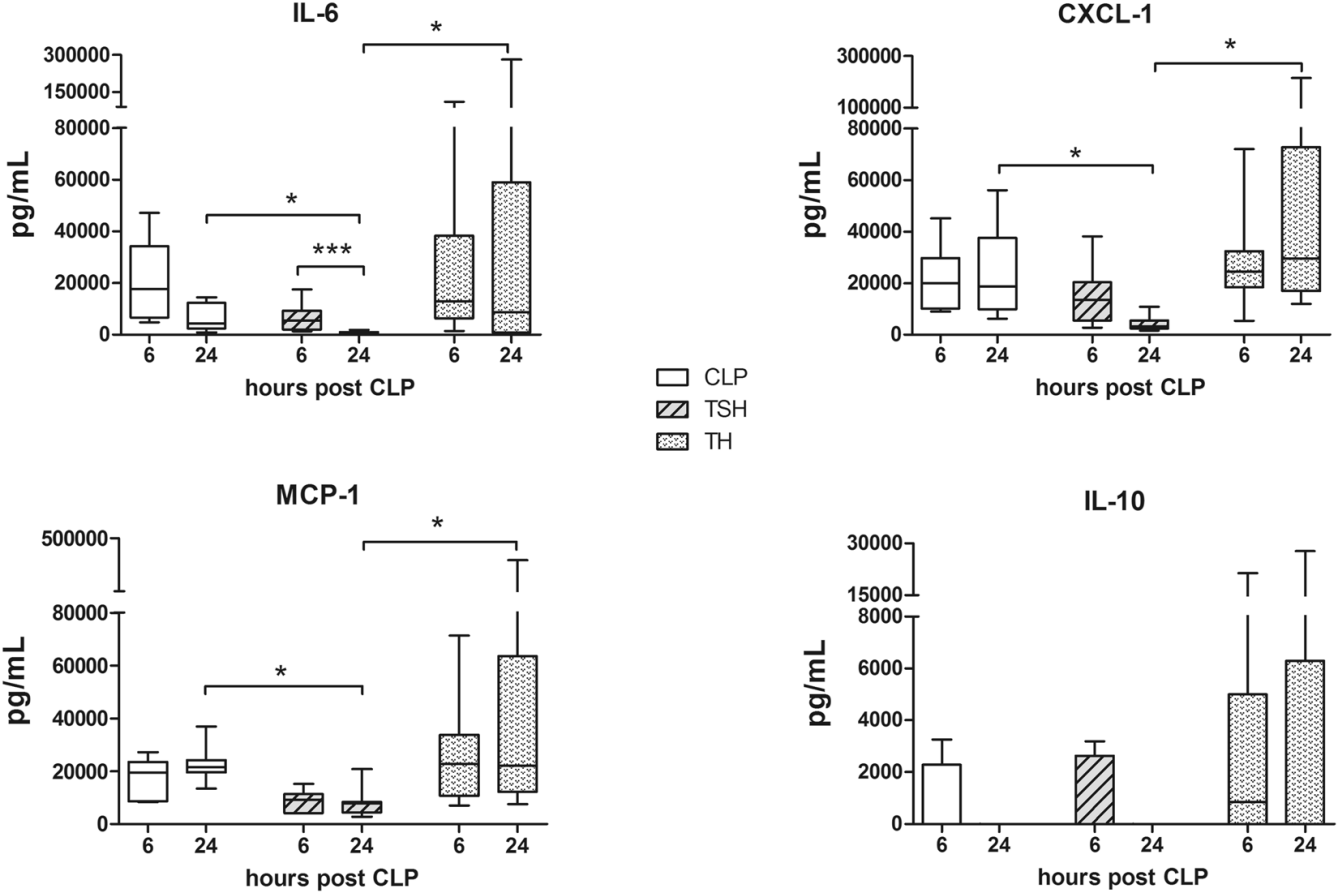
Selected plasma cytokines at 6 h and 24 h after TSH/TH-CLP. Female BALB/c mice were subjected to femur fracture, splenectomy and hemorrhagic shock (TSH) or femur fracture and hemorrhagic shock (TH) followed by cecal ligation and puncture (CLP) to induce polymicrobial abdominal sepsis 2 days later, or to CLP alone. Plasma levels of IL-6, IL-10, CXCL-1 and MCP-1 were assessed at 6 h and 24 h after TSH (TSH-CLP) or TH (TH-CLP) followed by secondary CLP or after CLP alone. For CLP: n = 7 per time point, TSH-CLP: n = 9 per time point, TH-CLP: n = 9 per time point. *p < 0.05.

Nine circulating cytokines, namely TNFα, IL-1β, IL-5, IL-6, IL-10, MIP-1α, MCP-1, CXCL-1, and IFNγ were analyzed at 6 and 24 h after CLP alone or as secondary hit after TH/TSH as a surrogate for the severity of the inflammatory response. As a general trend, TH as first hit was more likely to cause a robust cytokine release post-CLP when compared to TSH. Additional splenectomy significantly reduced the release at 24 h post-CLP compared to both TH-CLP and CLP alone in three out of nine cytokines, namely IL-6, CXCL-1 and MCP-1, i.e. by approx. 75- and 11-fold for IL-6, 12- and 5-fold for CXCL-1, and 3- and 8-fold for MCP-1. (Fig. 3).

### Part B

#### Pronounced leukocytosis and neutrophilia in mice subjected to polytrauma with splenectomy

**Figure 4:**
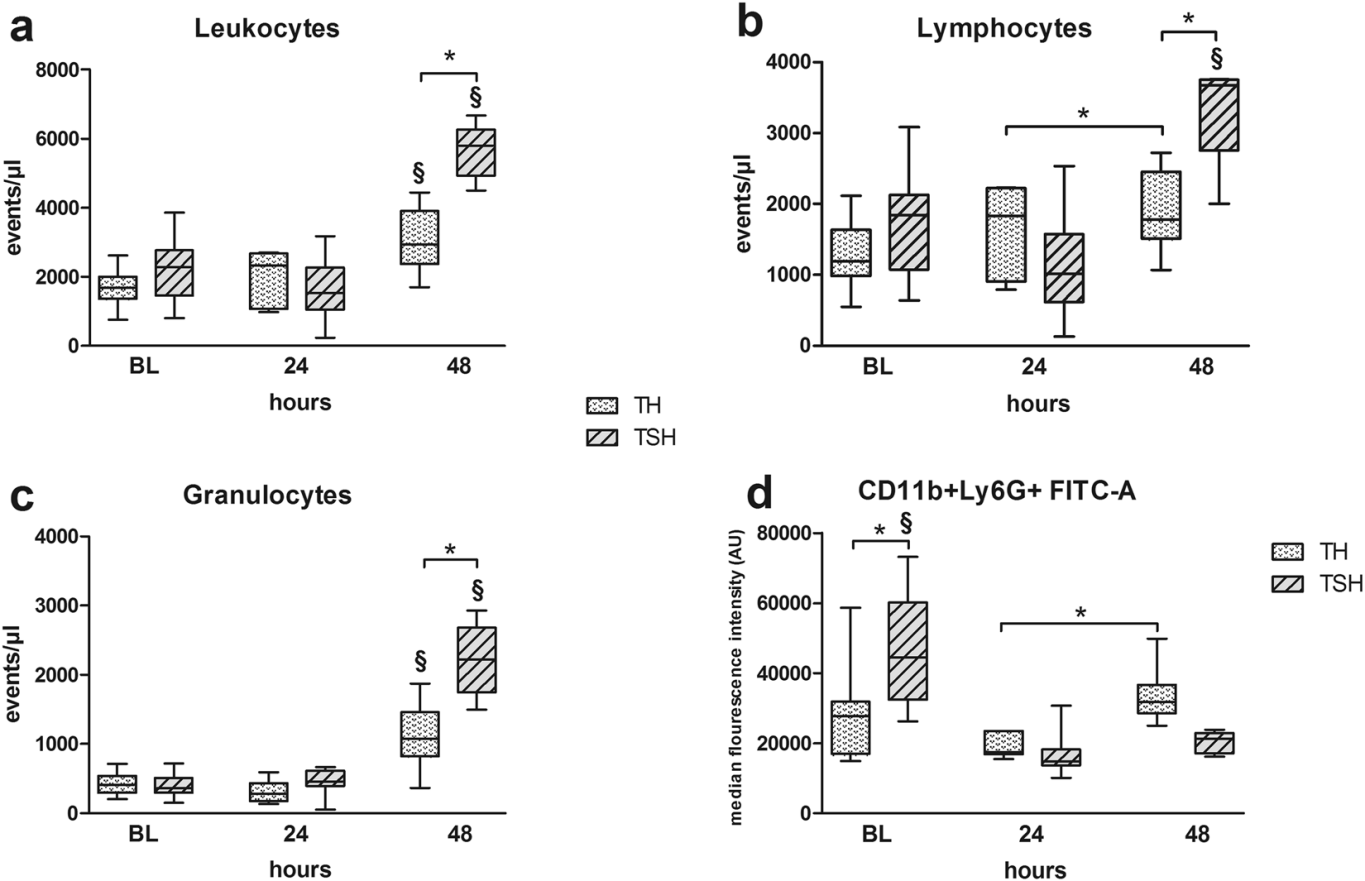
Comparison of leukocyte populations after TSH and TH. Female BALB/c mice were subjected to femur fracture, splenectomy and hemorrhagic shock (TSH) or femur fracture and hemorrhagic shock (TH). (**a**–**c**) Absolute numbers of circulating leukocytes, lymphocytes and granulocytes at BL (induction of trauma), 24 h and 48 h after TSH or TH. (**d**) Comparison of median fluorescence activity of activated granulocytes (CD11b+Ly6G+ cells) at BL (induction of trauma), 24 h and 48 h after TSH or TH. For TH: BL n = 24, 24 h n = 16, 48 h n = 12. For TSH: BL n = 11, 24 h n = 6, 48 h n = 5. *p < 0.05, ^§^p < 0.05 compared to all other time-points from the same group (i.e. TSH or TH).

Whenever appropriate, posttraumatic cellular parameters were measured in a 2-tier approach: (a) absolute number of events and/or (b) median fluorescence intensity. Both polytrauma models provoked leukocytosis (Fig. 4a) and neutrophilia (Fig. 4c) with more pronounced changes in mice subjected to TSH. At 48 h after trauma, circulating leukocytes were 1.8-fold, granulocytes 1.9-fold and lymphocytes 1.7-fold higher in TSH mice (Fig. 4b) compared to TH group (p < 0.05). Median fluorescence intensity of activated granulocytes (CD11b+Ly6G+) in TSH group dropped by 50% (p < 0.05) after trauma and was comparable in both groups at 24 h and 48 h (Fig. 4d).

#### MHC-2 expression decreased after TH but increased after TSH

**Figure 5:**
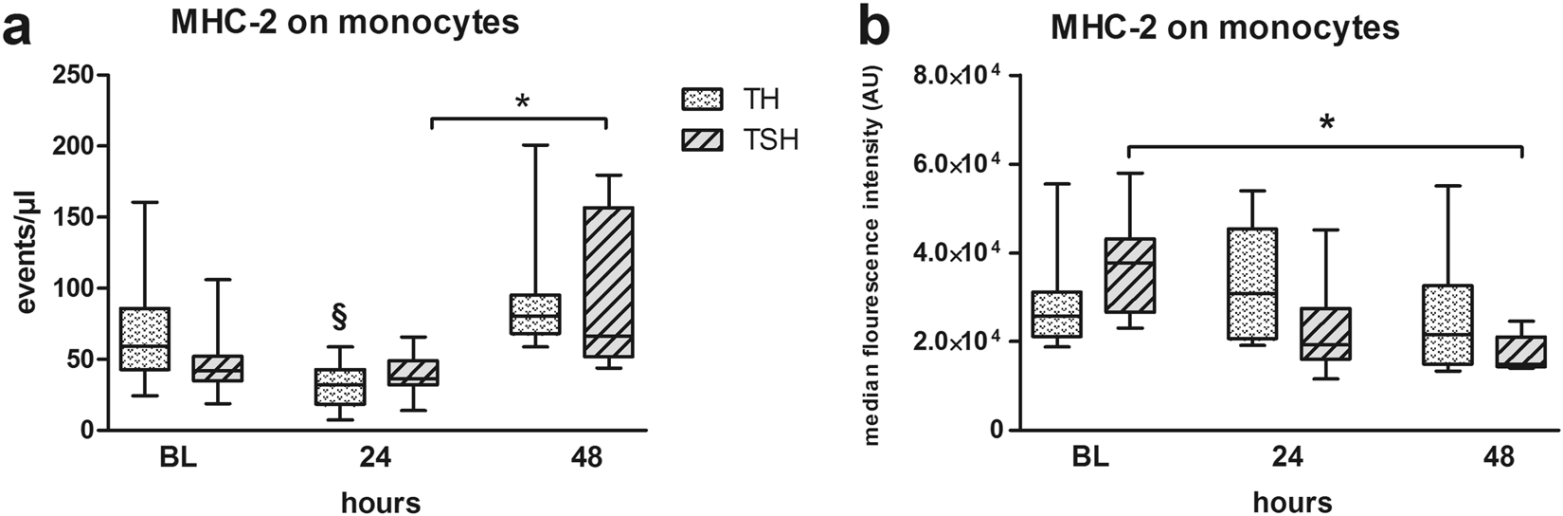
(**a**) Comparison of MHC-2+ monocytes. Female BALB/c mice were subjected to femur fracture, splenectomy and hemorrhagic shock (TSH) or femur fracture and hemorrhagic shock (TH). (**a**) MHC-2 expressing monocytes at BL (induction of trauma), 24 h and 48 h after TSH and TH. B. Median fluorescence intensity of MHC-2 expression on monocytes at BL (induction of trauma), 24 h and 48 h after TSH or TH. For TH: BL n = 7, 24 h n = 16, 48 h n = 12. For TSH: BL n = 11, 24 h n = 6, 48 h n = 5, *p < 0.05, ^§^p < 0.05 compared to all other time-points from the same group (i.e. TSH or TH).

Antigen presentation capacity of monocytes was assessed using MHC-2 expression. In TH mice, a 47% decrease (p < 0.05) of MHC-2-positive monocytes was detected at 24 h when compared to BL which recovered until 48 h (Fig. 5a). In contrast, TSH caused a 33% drop within 24 h, followed by a 3-fold increase of circulating MHC-2-expressing monocytes within 48 h (p < 0.05 compared to 24 h post TSH). Despite this increase, the median MFI of MHC-2 expression on monocytes was decreased significantly by 55% at 48 h post-TSH compared to baseline, signaling less activity of the respective cells while it remained unaltered in TH mice (Fig. 5b).

#### Polytrauma with splenectomy induced a rise in circulating T-cell populations

**Figure 6:**
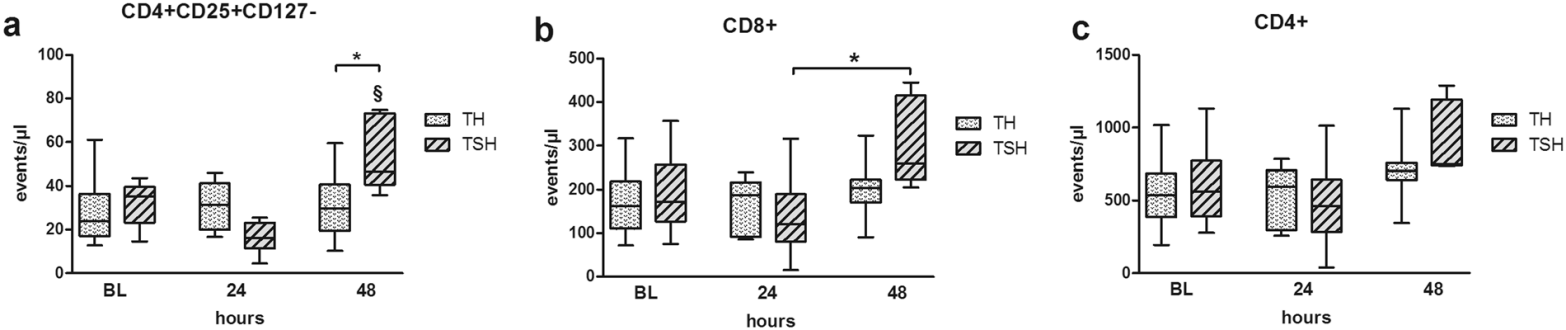
Comparison of T-cell populations. Female BALB/c mice were subjected to femur fracture, splenectomy and hemorrhagic shock (TSH) or femur fracture and hemorrhagic shock (TH). Absolute numbers of circulating (**a**). CD4+CD25+CD127− T-cells, (**b**). CD8+ T-cells and (**c**). CD4+ T-cells at BL (induction of trauma), 24 h and 48 h time point after TSH or TH. For TH: at BL n = 24, 24 h n = 16, 48 h n = 12. For TSH: BL n = 11, 24 h n = 6, 48 h n = 5. *p < 0.05.

TSH induced a 41% increase in circulating regulatory T-cells at 48 h compared to TH (p < 0.05) (Fig. 6a). Mice that underwent TSH also developed a 35% increase (p < 0.05) of absolute CD8+ T-cells within 48 h, while their CD4+ T-cell counts did not change after TH alone (Fig. 6b,c). The TH insult affected neither the circulating regulatory T-cells, nor the CD4+, or CD8+ T-cell counts (Fig. 6).

#### Bimodal cytokine response to LPS stimulation of mice subjected to polytrauma with and without splenectomy

**Figure 7:**
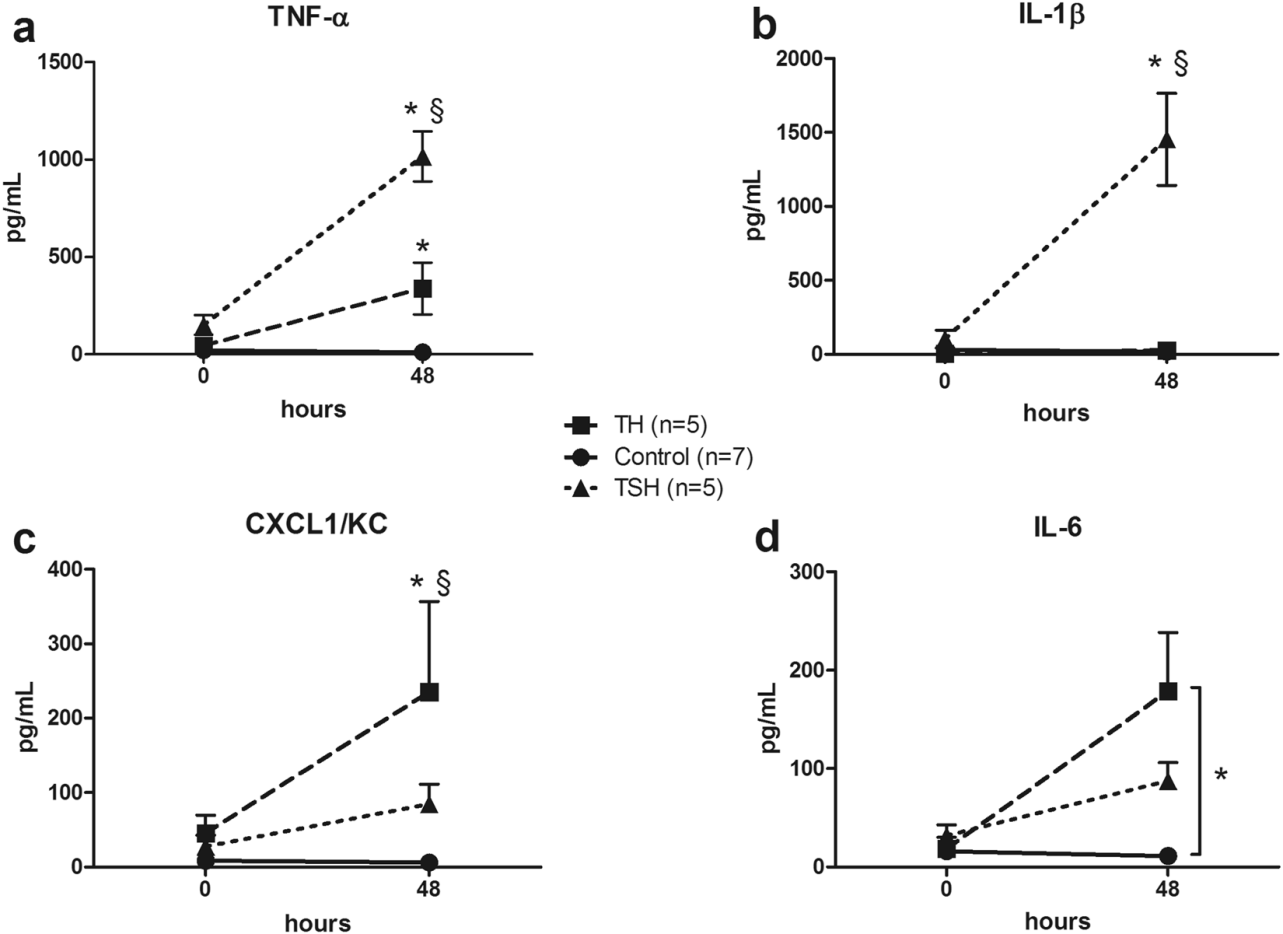
*Ex-vivo* cytokine release upon LPS stimulation of the whole blood. Female BALB/c mice were subjected to femur fracture, splenectomy and hemorrhagic shock (TSH) or femur fracture and hemorrhagic shock (TH) and whole blood was collected at BL and 48 h. (**a**–**d**) TNFα, IL-1β, IL-6 and CXCL-1 release upon whole blood stimulation with 10 µg LPS at 48 h after TH (square) or TSH (triangle) compared to healthy control group. (**a**–**c**) *p < 0.05 between trauma models, ^§^p < 0.05 compared to both groups. (**d**) *p < 0.05 Data presented as mean ± SD.

Stimulation of the whole blood with LPS at 48 h after trauma resulted in a stronger release of TNFα and IL-1ß in TSH animals. TNFα concentration was higher by approx. 94-fold and 10-fold compared to Control and TH group, while IL-1ß accumulation increased by approx. 50-fold compared to TH (p < 0.05) (Fig. 7a,b). In contrast, TH caused a more pronounced IL-6 (by 15-fold compared to Control group) and CXCL-1 accumulation (by 2.7-fold compared to TSH) (Fig. 7c,d).

#### Additional splenectomy increased the circulating complement component C5a

**Figure 8:**
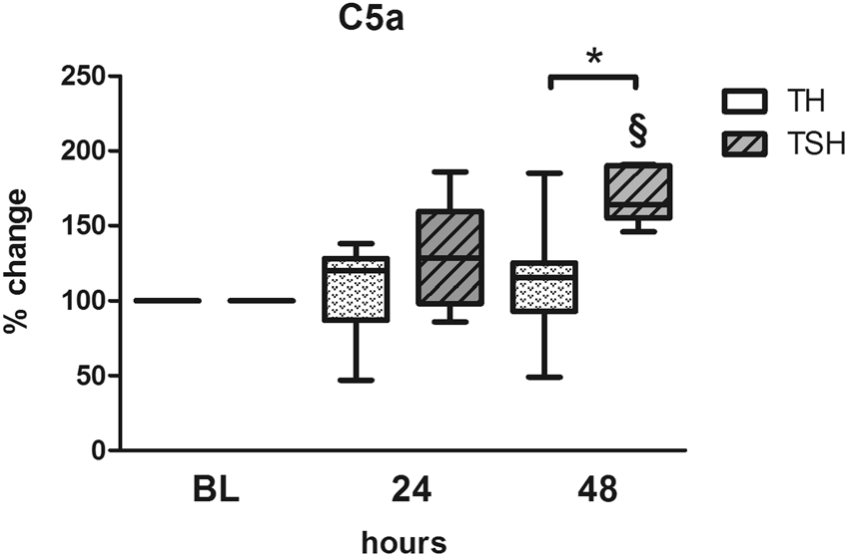
Complement Component C5a. Female BALB/c mice were subjected to femur fracture, splenectomy and hemorrhagic shock (TSH) or femur fracture and hemorrhagic shock (TH) and whole blood was collected at BL, 24 and 48 h to assess C5a levels from 1:10 diluted plasma samples using ELISA. For TSH at BL n = 12, at 24 h n = 12, at 48 h n = 5, for TH n = 11 for all time points. *p < 0.05.

TSH but not TH insult resulted in a relative increase of circulating complement component C5a. At 48 h post trauma, C5a level was approx. 70% higher in mice that underwent TSH when compared to baseline (p < 0.05). Furthermore, at 48 h after trauma, C5a relative concentration was 1.5 fold higher in TSH animals compared to TH (p < 0.05) (Fig. 8).

#### Only polytrauma with splenectomy stimulated phagocytosis

**Figure 9:**
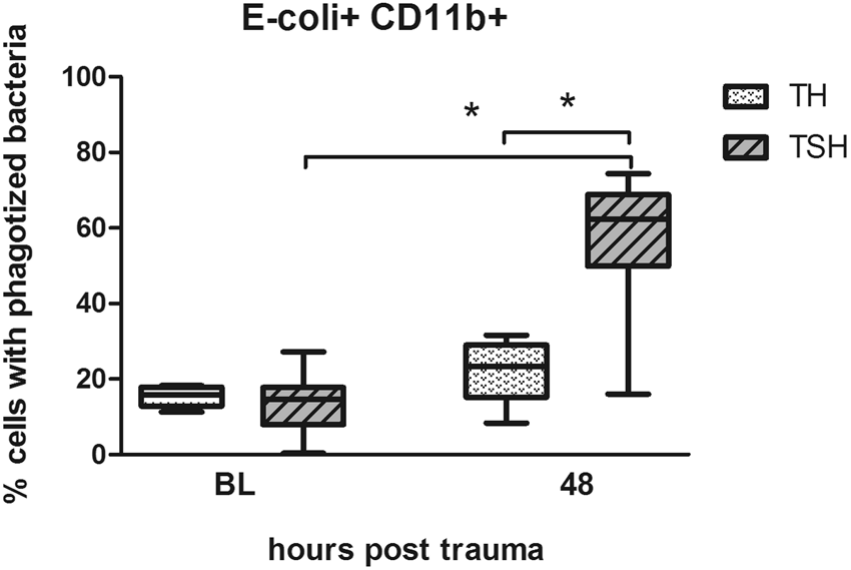
Phagocytosis assay. Female BALB/c mice were subjected to femur fracture, splenectomy and hemorrhagic shock (TSH) or femur fracture and hemorrhagic shock (TH) and whole blood was collected at BL and 48 h. Phagocytic uptake of pHrodo® labeled *E. coli* by CD11b+ cells was detected at baseline and 48 h after TSH and TH when incubated for 24 h at 0 °C. For TSH at BL n = 19, at 48 h n = 8, for TH n = 8 for both time points. *p < 0.05.

At 48 h post-trauma, the TSH insult led to a 4-fold increase in phagocytic capacity of peritoneal macrophages and granulocytes when compared to baseline. In contrast, phagocytosis was not enhanced in the cells isolated from mice subjected to TH (Fig. 9). Direct inter-group comparison showed that accumulation of cells containing PHrodo® *E. coli* BioParticles® was 2.6-fold higher in the TSH group compared to TH.

#### M1/M2 macrophage polarization dynamic was similar in both TH and TSH

**Figure 10:**
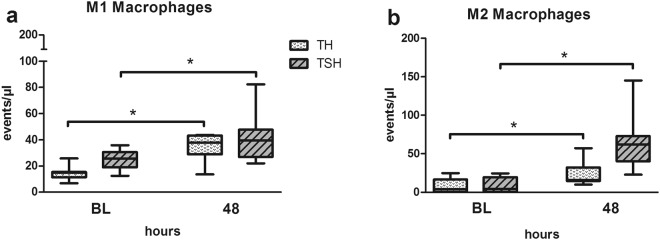
Macrophage polarization. Female BALB/c mice were subjected to femur fracture, splenectomy and hemorrhagic shock (TSH) or femur fracture and hemorrhagic shock (TH) and whole blood was collected at BL and 48 h to investigate changes in the macrophage polarization. (**a**) M1 macrophages, (**b**) M2 Macrophages. For TSH n = 8 and for TH n = 7 for both time points. *p < 0.05.

Independent of the additional splenectomy, both trauma insults had similar effects on the M1/M2 macrophage polarization profile. At 48 h after trauma, when compared to baseline, M1 macrophages were markedly increased by 2.3 and 1.6 fold (Fig. 10a) and M2 macrophages by 3.3 and 7.4 fold (Fig. 10b) in TH and TSH, respectively (p < 0.05). However, no inter-group differences between TH and TSH group were detected.

## Discussion

This study shows that compared to hemorrhagic-traumatic shock alone (TH), BALB/c mice subjected to traumatic-hemorrhagic shock combined with splenectomy (TSH) underwent a more robust modulation of the innate and adaptive immune responses and had improved survival after secondary sepsis.

Clinical studies have repeatedly associated polytraumatic injuries with an unequivocally harmful dysregulation of the immune response^41,42^. However, the most recent findings suggest that this phenomenon is much more heterogeneous. Polytrauma in general, irrespective of splenectomy, decreased the antigen presenting capacity in human patients^2,42^. The large 2017 study showed that polytraumatized patients had better survival after (secondary) infection with Gram-negative bacteria compared to the infected but non-traumatized subjects^43^. The same authors demonstrated that mice subjected to a polytrauma-model (unilateral pneumothorax and femur fracture) followed by *P. aeruginosa* infection also survived better than those without trauma; they concluded that the initial trauma may generate a tolerance to alarmins by a beneficial modulation of immune reactions when challenged with secondary bacterial infection.

Similarly to studies that generally reported detrimental effects of polytrauma^2,41^, splenectomy in trauma patients was also repeatedly associated with an increased risk for acute secondary infections such as pneumonia and delayed immunologic impairments^2,11–15,41^. In a 1983 study, approximately 23% of the 503 splenectomized trauma patients (who survived the first 10 days after trauma) developed sepsis^44^. Yet, the largest to date retrospective analysis of 28,002 US trauma patients with spleen injury contrasts that: splenectomized (vs. non-splenectomized) subjects had a significantly decreased length of hospital stay, ventilator and ICU days, and the incidence of acute respiratory distress syndrome was not higher^45^. Our current mouse study is in line with the latter study. The data demonstrate that improvement of survival can be attributed to the beneficial modulation of the immuno-inflammatory response after polytrauma. The spleen serves as a major reservoir of cytokine producing monocytes/macrophages^46^. We found that TSH radically attenuated the release of IL-6, CXCL-1 and MCP-1 in the secondary CLP-induced sepsis but TH alone did not. In two rat studies with traumatic brain injury^47^ and ischemia-reperfusion induced acute lung injury^48^ model, splenectomy had a similar effect of decreasing circulating^47^ and pulmonary cytokines^48^. The post-CLP attenuation of IL-6 release *in vivo* we observed is consistent with the improvement of survival given that IL-6 elevation accurately predicts death (and its decline predicts survival) in pre-and clinical sepsis^49,50^. In another (single hit) mouse CLP study, an improvement of survival after splenectomy was associated with a decline in circulating HMGB-1^51^. We also verified to what extent splenectomy influenced the *ex-vivo* capacity of the circulating blood cells to release cytokines after trauma. Interestingly, the response was diverse: IL-6 and CXCL-1 were lower, while TNF-α and IL-1β formation capacity of circulating cells was higher in TSH mice (compared to TH). To gain a deeper insight, we also examined multiple cellular markers typically used for monitoring of immune status/capacity in the ICU patients^52^. Although both types of trauma induced a clear neutrophilia, the cellular activation of granulocytes (i.e. CD11b expression) was only dampened in TSH mice. This was simultaneous with a decrease of the MHC-2 median fluorescence intensity on monocytes at day 2 post-TSH, which is suggestive of a decreased antigen presentation capacity. However, the similar M1-to-M2 polarization dynamics in the macrophage phenotype after both TH and TSH did not attest to that. Additionally, immunosuppressive regulatory T-cell count was elevated alongside with an increase of complement component C5a production but only after TSH, and this coincided with an increased phagocytic capacity of granulocytes and monocytes in the TSH mice. Mollnes *et al*. showed that blockage of C5a receptor inhibited phagocytosis *in vitro*^53^ which could implicate a stimulating effect of C5a on phagocytotic capacity. Overall, these findings show that splenectomy caused an additional, multifaceted modulation of the TH-induced immuno-inflammatory response which effectively counteracted the harmful reactions triggered by CLP sepsis two days later.

It is however unclear whether the altered immune-inflammatory response after splenectomy can be attributed to the elimination of the robust cytokine-producing monocytes/macrophages or other type of signaling cascade modifications (given that phagocytic capacity was enhanced not impaired). Similar beneficial effects of splenectomy were seen in rat traumatic brain injury (TBI) and heart and lung ischemia reperfusion injury models^47,48,54^. It remains to be established whether the observed benefits are, and to what extent, based on some immuno-inflammatory denominators common for all post-traumatic response types. In the context of CLP sepsis, the (splenectomy-dependent) survival advantage does not appear to be time-dependent, at least within the short time-frame; Huston *et al*.^51^ performed splenectomy simultaneously with CLP (versus 2 days prior to CLP in the current study). Of note, only one animal study to date demonstrated detrimental effects of splenectomy (combined with an insult); it exacerbated lung injury and heightened inflammatory response in BALB/c mice with acute ischemic kidney injury^22^.

The current study also alerts to another vital issue: the need for verification of an initial polytrauma hit (and its elements) on the secondary hit of interest (CLP in this study). Combinations of different trauma elements (e.g. fracture, splenectomy, cecostomy, hemorrhage, and laparotomy) should not be chosen reflexively; individual elements have divergent modulatory capacity and can produce different injury-specific inflammatory patterns^26,28,29^. Construction of a 2-hit model that replicates clinical situations has to acknowledge the fact that the murine immune system is more resistant compared to humans^31,55^. In human patients, septic complications typically develop in the first days after the initial trauma^56^; in mice, they have to be artificially created. In this context, we caution against an unreflective adaptation to mice the early-onset timeline (typically 2–8 days^57–59^) of secondary human infection/sepsis. Although there are similarities in the temporal inflammatory^60,61^ and genomic host response^62^ patterns between humans and mice, those dynamics may vary depending on the injury type and other variables such as outbred/inbred strain, rodent age etc.^63^. Thus, in a clinically-relevant 2-hit model, not the day span but the similarity of the immuno-inflammatory blueprints (i.e. between animal and patient) developing after polytrauma should dictate the timing of the hits. Furthermore, characterization of the post-traumatic immuno-inflammatory phenotypes has been typically based on limited resources; mainly blood/plasma and *ex-vivo* stimulation of cells isolated from various tissues^64–66^. In any polytrauma model, assessment of the degree and true impact of given immune dysregulations based on changes in the peripheral blood and/or cells from few organs/tissues can be misleading. Strong differences among compartments are likely^67^ and the blood compartment constitutes only a small representation of the entire immuno-inflammatory status of the host. Our results strongly indicate that accompanying survival studies (or using a reliable death surrogates^68,69^) should be viewed as an important validation step in 2-hit modeling systems. Lack of such a verification may lead to misleading results from which inappropriate conclusions are drawn.

This study has several limitations. In all experiments, only young healthy female mice were used; while they represent an important cohort of adult fertile women, they do not account for other frequently encountered variables such as age, chronic comorbidities, male gender. We did not expand the trauma-CLP gap beyond 48 h. The current design was purposely chosen given that we sought to investigate the 24-to-48 h delay span, which is the most frequently used timeline in two-hit models featuring secondary sepsis^25,35,37,70^. For similar reasons, the study did not investigate the immune status in other compartments except the blood. While the circulatory compartment is the easiest accessible and therefore most frequently used data source in pre-and clinical practice, changes at different sites can be equally informative and influential.

## Conclusions

Splenectomy strongly affected the acute immuno-inflammatory response to polytrauma and secondary sepsis by dampening antigen presentation capacity, the release of specific proinflammatory mediators and granulocyte activation but not phagocytic capacity. This modulation resulted in a strong increase of survival in splenectomized mice after secondary CLP sepsis, contrasting the increased mortality by polytrauma alone (without splenectomy). Additionally, our findings alert to two specific aspects of polytrauma modeling: (1) individual elements that build a polytrauma model (e.g. splenectomy, hemorrhage, fractures, brain injury) can differentially modulate the immune responses of the secondary hit, (2) an impact of the first polytrauma hit should not be solely judged by the humoral/cellular phenotype from a single or two compartments (e.g. blood) but by the outcomes of the secondary hit (e.g. sepsis).

## Electronic supplementary material

Dataset 1

## Data Availability

The raw data supporting the conclusions of this manuscript will be made available by the authors, without undue reservation, to any qualified researcher.
