# Supplementary material for: Splenectomy modulates early immuno-inflammatory responses to trauma-hemorrhage and protects mice against secondary sepsis
Source: Sci Rep. 2018 Oct 5;8:14890. doi: 10.1038/s41598-018-33232-1 (PMC6173732; doi:10.1038/s41598-018-33232-1)
Supplement: Supplementary file 1 — Dataset 1 [file 41598_2018_33232_MOESM1_ESM.pdf]

Splenectomy modulates early immuno-inflammatory responses to trauma-hemorrhage and protects mice against secondary sepsis

S. Drechsler[1], P. Rademann[1,2], J. Zipperle[1], M. Jafarmadar[1], A. Klotz[1], S. Bahrami[1], M.F. Osuchowski[1]

[1] Ludwig Boltzmann Institute for Experimental and Clinical Traumatology in the AUVA Research Center , Vienna, Austria

[2] Current Address: Center for Experimental Medicine, Medical Faculty, University of Cologne, Cologne, Germany

Supplementary Figure 1

a

Gating strategy for flow cytometric analysis of peripheral leukocytes. Representative gating strategy for leukocyte subsets with an emphasis on (a) Lymphocytes and (b) Monocytes. Diluted peripheral whole blood was subjected to RBC lysis and divided into a Lymphocyte and Monocyte panel. Lymphocytes (**a**): A CD4+CD8a- population was gated from all captured events and further assessed for the positivity for CD25. Using fluorescence-minus-one (FMO) stainings, the respective population was then plotted against CD127 to identify a CD4+CD25+CD127- subset.
Monocytes (**b**): All captured events were analyzed for the presence of CD11b and Ly6G and were divided into CD11b+Ly6Ghigh, CD11b+Ly6Glow and CD11b-Ly6G-. The defined populations were then regated in dot blots with fluorescence channels for MHCII and CD11b versus side scatter (SSC). In addition, simultaneous stainings with CD11b and F4/80 were carried out to confirm gating based on CD11b signal versus SSC. Fluorescence from a specific antigen is given as mean fluorescence intensity (MFI) from the respective conjugate. Absolute counts of identified events were calculated per volume and given as events/µL.

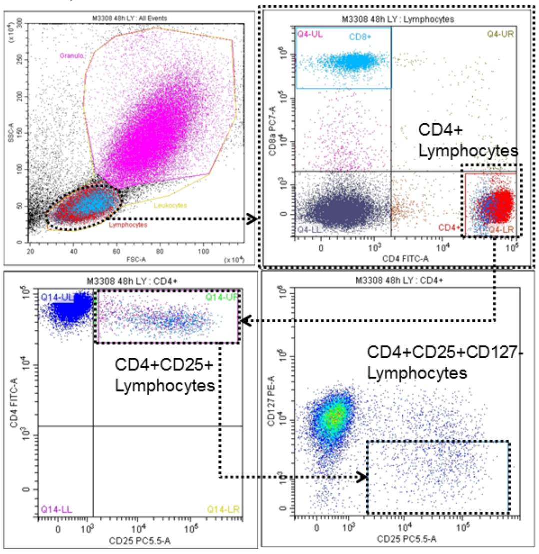

b

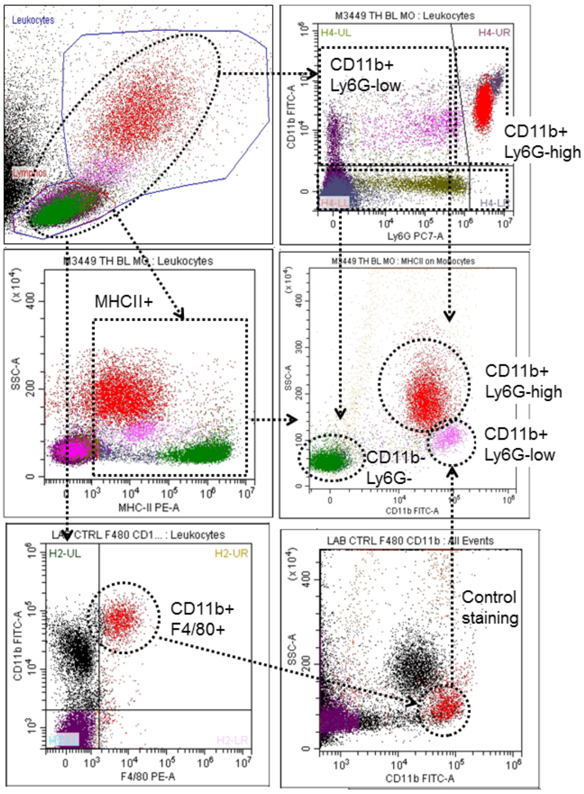

Supplementary Figure 2

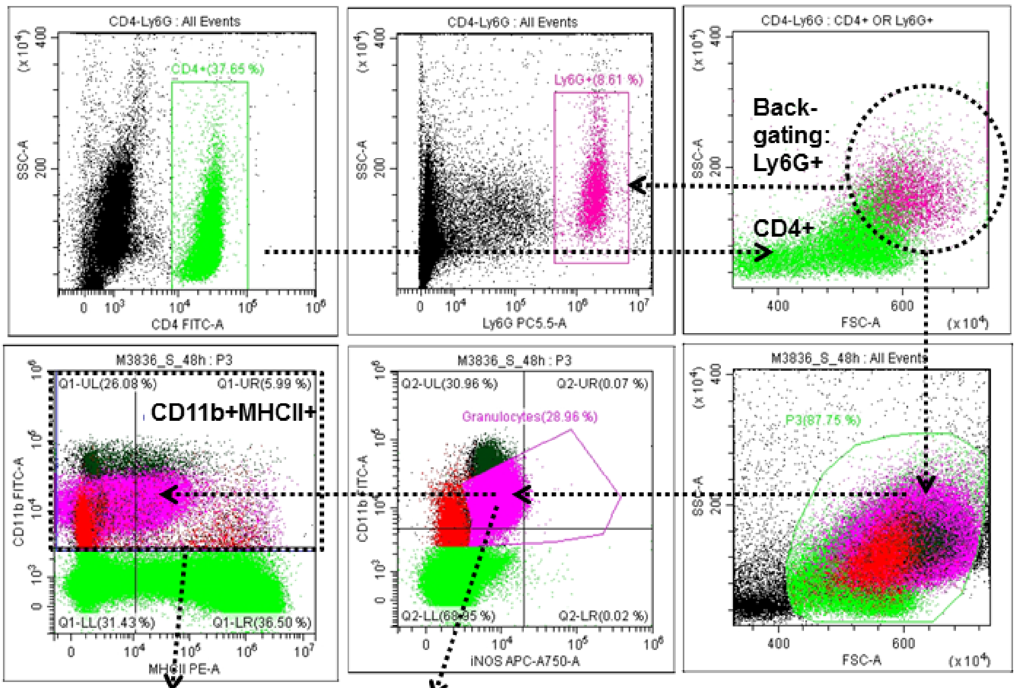

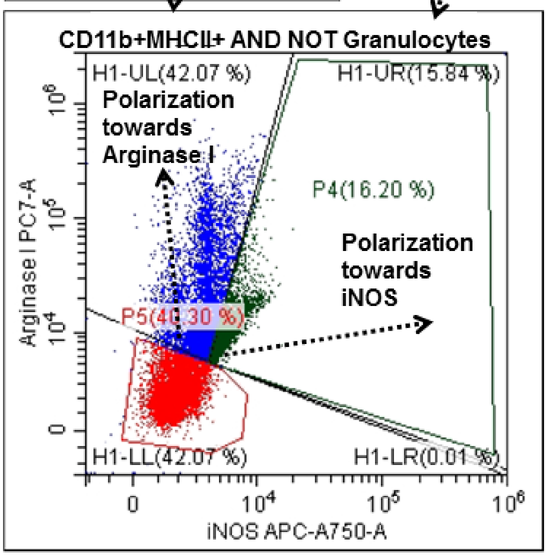

Flow cytometric analysis of macrophage polarization in peripheral blood. Representative gating strategy for monocyte-derived macrophages and the polarization towards Arginase I and inducible NO synthase (iNOS) metabolism. Peripheral blood was stained with antibodies targeting extracellular antigens (CD11b, MHCII or Ly6G, CD4), fixed, permeabilized and subsequently stained for the intracellular antigens Arginase I and iNOS. Lymphocytes and Granulocytes were identified based on positivity/ negativity for CD4 and Ly6G and morphology in the FSC-SSC. A combinating gate with an exclusion logic for granulocytes was defined based on positivity for CD11b, MHCII and iNOS. The respective subpopulation was then plotted in an iNOS versus Arginase I window and the expansion of the population towards increased Arginase I or iNOS expression was defined as polarized activation.
